# Supplementary figures and images for: Compositional editing of extracellular matrices by CRISPR/Cas9 engineering of human mesenchymal stem cell lines
Source: eLife. 2025 Mar 28;13:RP96941. doi: 10.7554/eLife.96941 (PMC11952750; doi:10.7554/eLife.96941)

# 27/6 RUNX2

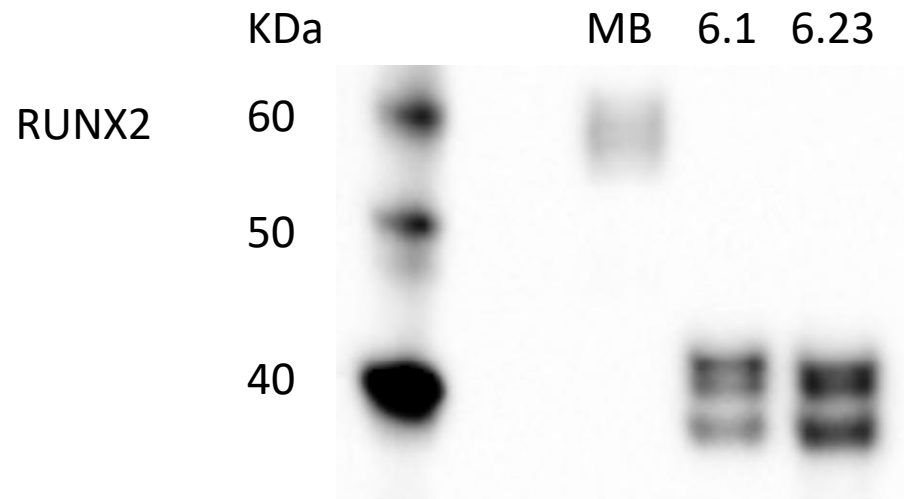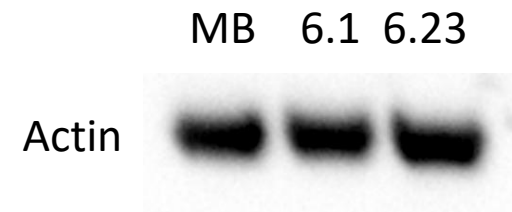

# 27/6 RUNX2 Original blots

Original plot: overexposed to highlight membranes

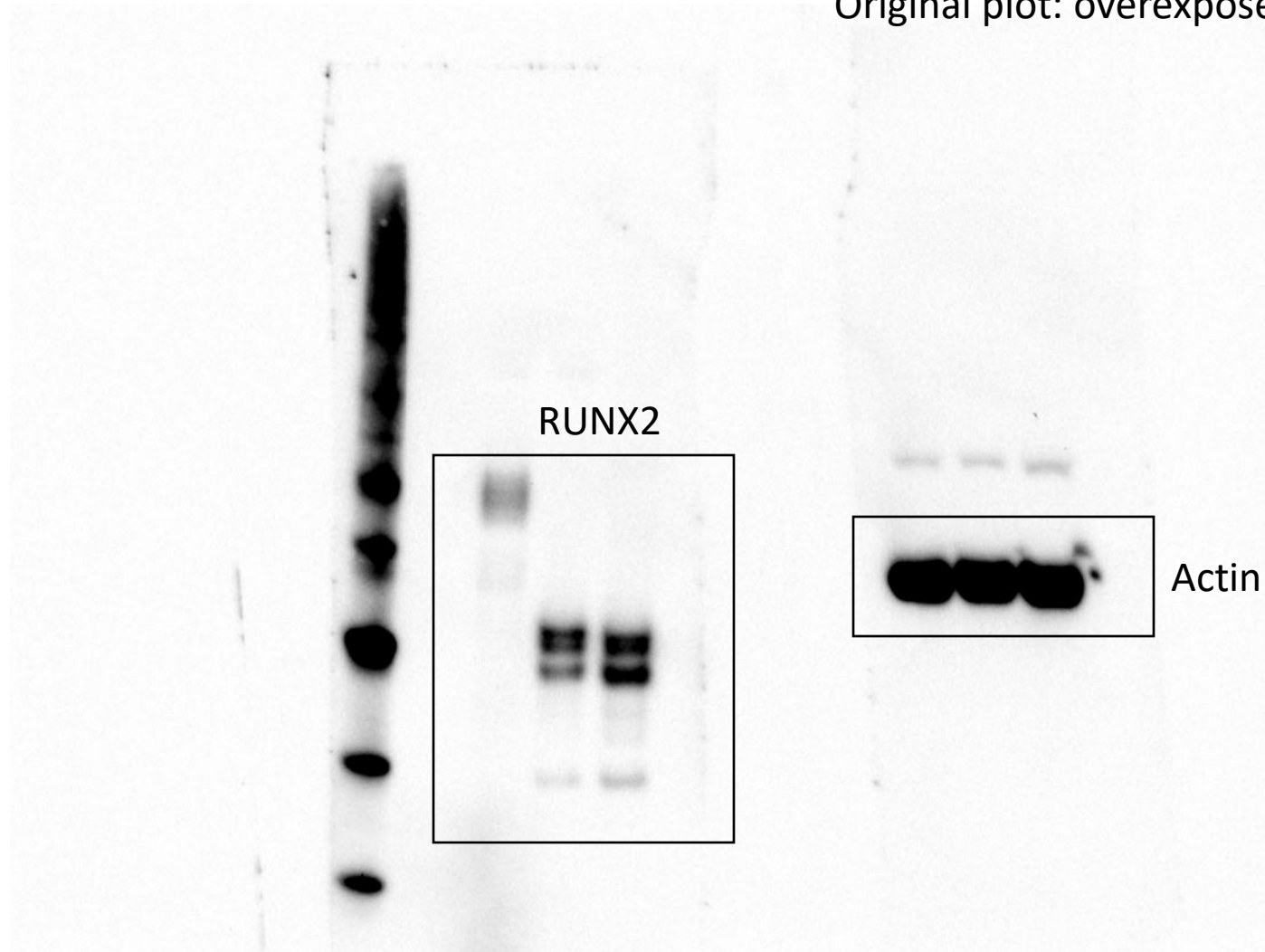

Supplement: Figure 3—source data 1. — Western blot analysis of RUNX2 in cultured mesenchymal sword of Damocles bone morphogenetic type-2 (MSOD-B) (MB) and RUNX2-edited cells (clones 6.1 and 6.23, respectively). The genetic editing of RUNX2 is confirmed by the detection of the truncated proteins. Actin is used as a control to normalize the protein content. Exposure was set at 13.7 s. [file elife-96941-fig3-data1.pdf]

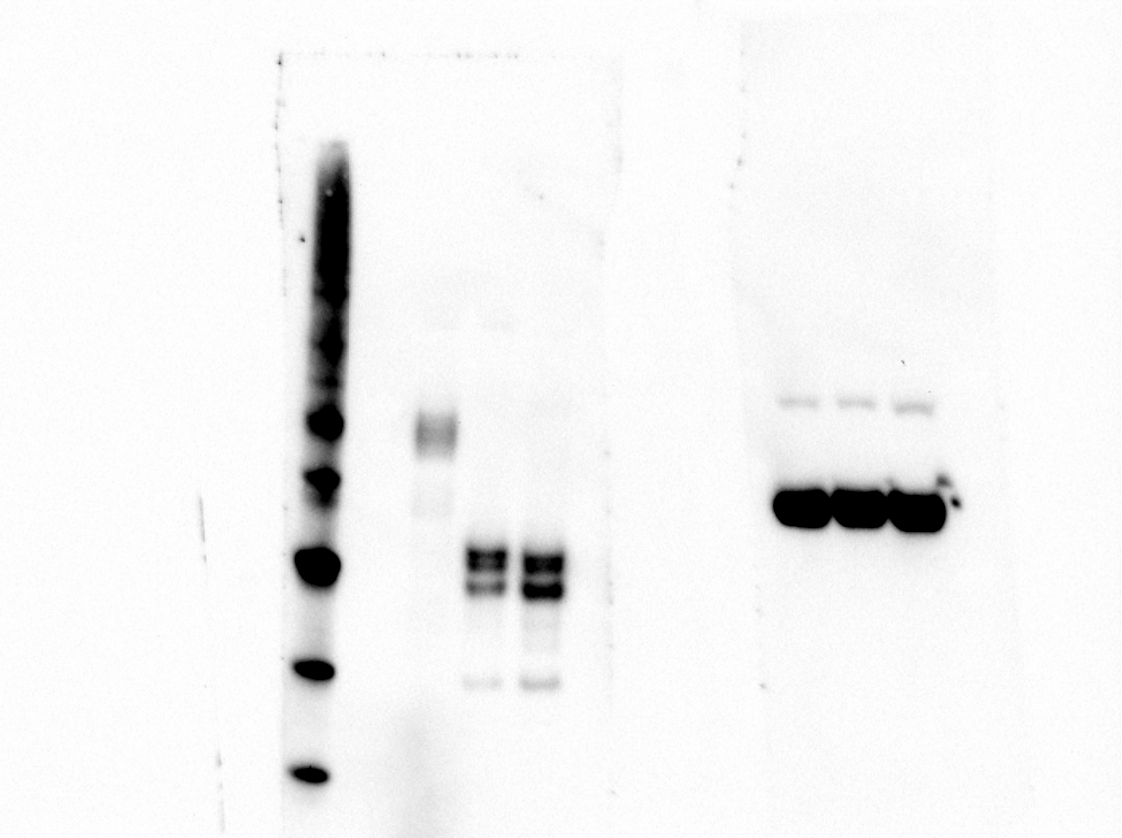

Supplement: Figure 3—source data 2. — Non-annotated western blot pictures of RUNX2 (left part of the gel) in cultured mesenchymal sword of Damocles bone morphogenetic type-2 (MSOD-B) (line 3) and RUNX2-edited cells (clone 6.1 line 4 and clone 6.23 line 5, respectively). The genetic editing of RUNX2 is confirmed by the detection of the truncated proteins. Actin (right part of the gel) is used as a control to normalize the protein content. [file elife-96941-fig3-data2.zip › _Exposure_13.7sec.tif]

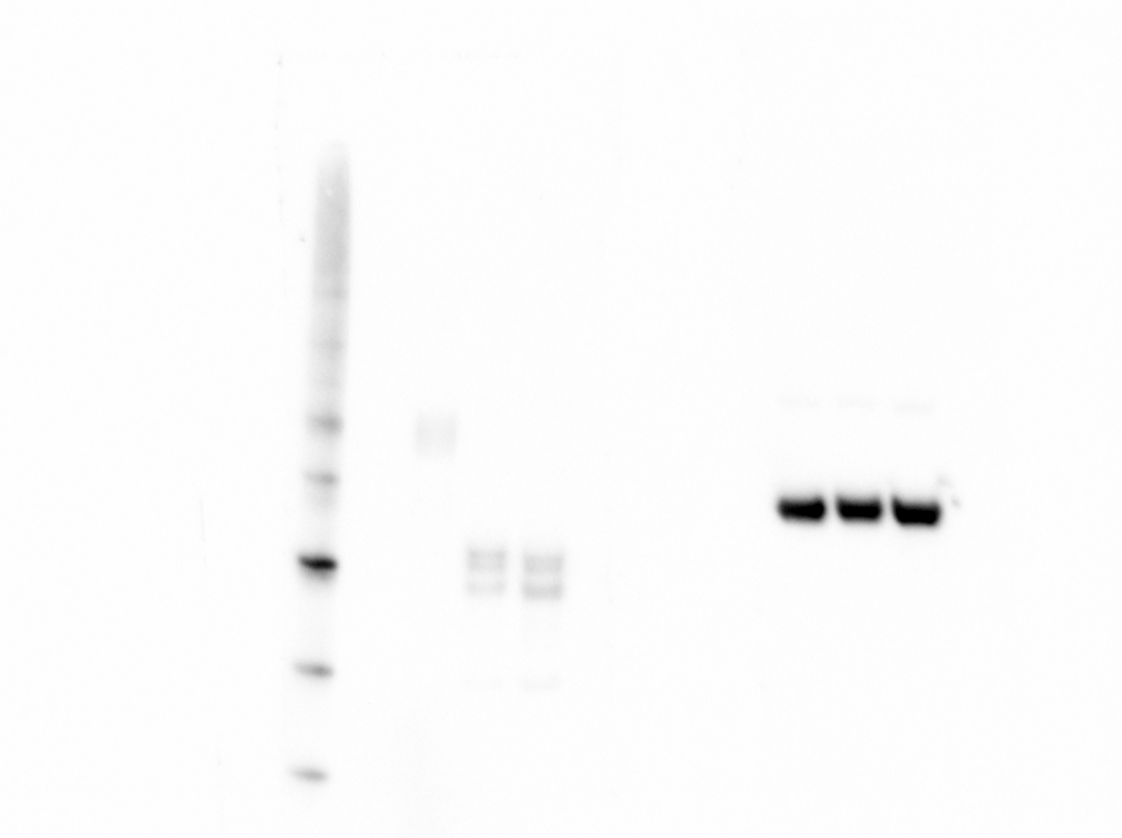

Supplement: Figure 3—source data 2. — Non-annotated western blot pictures of RUNX2 (left part of the gel) in cultured mesenchymal sword of Damocles bone morphogenetic type-2 (MSOD-B) (line 3) and RUNX2-edited cells (clone 6.1 line 4 and clone 6.23 line 5, respectively). The genetic editing of RUNX2 is confirmed by the detection of the truncated proteins. Actin (right part of the gel) is used as a control to normalize the protein content. [file elife-96941-fig3-data2.zip › Actin source file_Exposure_3.1sec.tif]

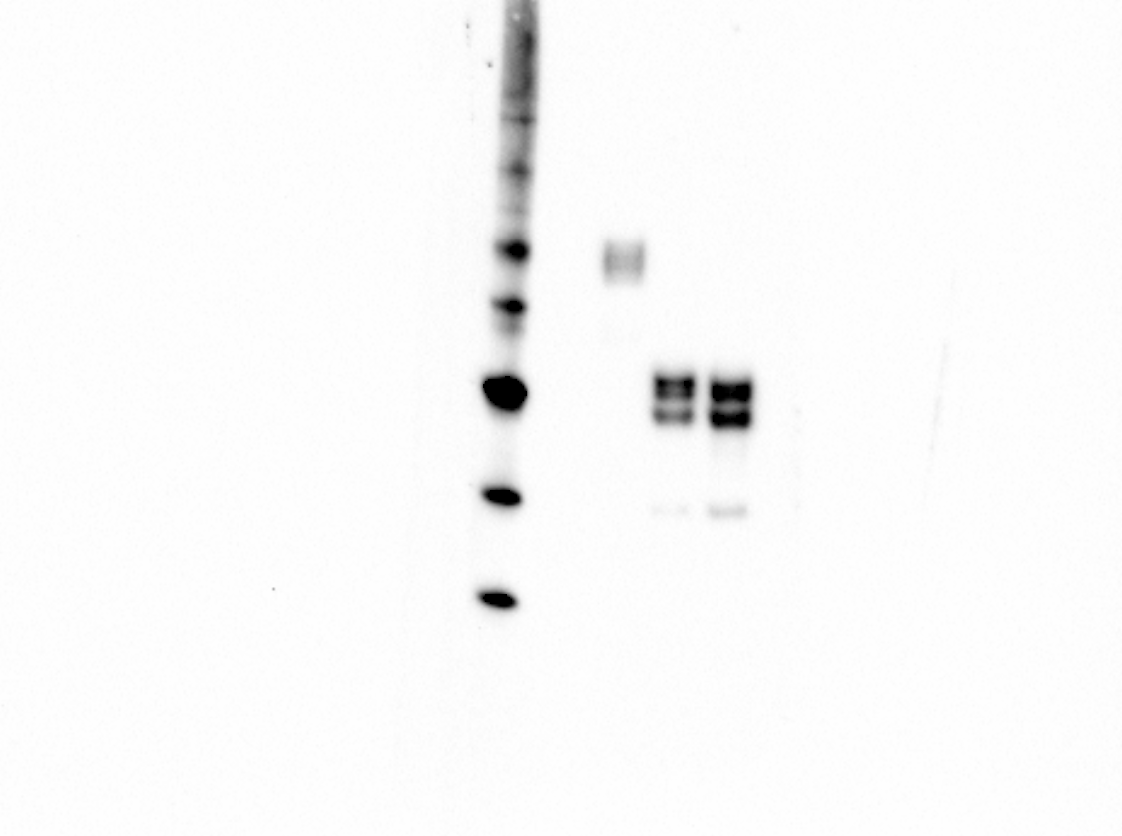

Supplement: Figure 3—source data 2. — Non-annotated western blot pictures of RUNX2 (left part of the gel) in cultured mesenchymal sword of Damocles bone morphogenetic type-2 (MSOD-B) (line 3) and RUNX2-edited cells (clone 6.1 line 4 and clone 6.23 line 5, respectively). The genetic editing of RUNX2 is confirmed by the detection of the truncated proteins. Actin (right part of the gel) is used as a control to normalize the protein content. [file elife-96941-fig3-data2.zip › RUNX2 in paper_Exposure_21.0sec.tif]
