# Supplementary material for: Compositional editing of extracellular matrices by CRISPR/Cas9 engineering of human mesenchymal stem cell lines
Source: eLife. 2025 Mar 28;13:RP96941. doi: 10.7554/eLife.96941 (PMC11952750; doi:10.7554/eLife.96941)
Supplement: Supplementary file 1. — Semiquantitative analysis utilizing a modified histological grading system, including parameters such as cellular morphology, matrix staining, surface regularity, thickness of cartilage, subchondral bone formation, and integration of adjacent cartilage, resulting in a comprehensive assessment of tissue regeneration and integration [file elife-96941-supp1.docx]

| **Scoring Rate (%) (SD)** | | | | | | |
| --- | --- | --- | --- | --- | --- | --- |
|  | Cell Morphology | Matrix Staining | Surface Regularity | Thickness of Cartilage | Regenerated Subchondral Bone | Integration with Adjacent Cartilage |
| **Healthy** | 100 (0) | 100 (0) | 100 (0) | 100 (0) | 100 (0) | 100 (0) |
| **MSOD-B** | 33,33 (28,86) | 16,66 (14,43) | 50 (0) | 8,33 (14,33) | 16,66 (14,33) | 33,33 (14,33) |
| **MSOD-B ΔR1** | 66,66 (14,33) | 50 (0) | 41,66 (14,33) | 33,33 (28,86) | 25 (25) | 50 (0) |
