# Supplementary material for: Compositional editing of extracellular matrices by CRISPR/Cas9 engineering of human mesenchymal stem cell lines
Source: eLife. 2025 Mar 28;13:RP96941. doi: 10.7554/eLife.96941 (PMC11952750; doi:10.7554/eLife.96941)
Supplement: Supplementary file 2. — The histological graded system is modified and adapted from previously established model as mentioned in Materials and methods section. This modified scale incorporates six distinct parameters to comprehensively evaluate the quality of tissue repair. [file elife-96941-supp2.docx]

| **Category** | **Point** |
| --- | --- |
| **Cell morphology** | |
| **Hyaline cartilage** | 4 |
| **Mostly hyaline cartilage or fibrocartilage** | 3 |
| **Mostly fibrocartilage** | 2 |
| **Mostly non-cartilage** | 1 |
| **Non-cartilage** | 0 |
| **Matrix-staining (metachromasia)** | |
| **Normal** | 3 |
| **Slightly reduced** | 2 |
| **Markedly reduced** | 1 |
| **No metachromatic staining** | 0 |
| **Surface regularity^a^** | |
| **Smooth** | 3 |
| **Moderate** | 2 |
| **Irregular** | 1 |
| **Severely irregular** | 0 |
| **Thickness of cartilage (%)^b^** | |
| **121–150** | 1 |
| **81–120** | 2 |
| **51–80** | 1 |
| **0–50** | 0 |
| **Regenerated subchondral bone** | |
| **Good** | 2 |
| **Moderate** | 1 |
| **Poor** | 0 |
| **Integration with adjacent cartilage** | |
| **Both edges integrated** | 2 |
| **One edge integrated** | 1 |
| **Neither edge integrated** | 0 |
| **Total maximum** | 16 |

- ^a^Total smooth area of the reparative cartilage compared with the entire area of the cartilage defect.
- ^b^ Average thickness of the reparative cartilage compared with that of the surrounding cartilage.
